# Laparoscopic surgery produced less surgical smoke and contamination comparing with open surgery: the pilot study in fresh cadaveric experiment in COVID-19 pandemic

**DOI:** 10.1186/s12893-021-01432-8

**Published:** 2021-12-16

**Authors:** Voraboot Taweerutchana, Tharathorn Suwatthanarak, Asada Methasate, Thawatchai Akaraviputh, Jirawat Swangsri, Chainarong Phalanusitthepha, Atthaphorn Trakarnsanga, Thammawat Parakonthun, Nicha Srisuworanan, Thikhamporn Tawantanakorn, Rosarin Ratanalekha, Varut Lohsiriwat, Vitoon Chinswangwatanakul

**Affiliations:** 1 Division of General Surgery, Office of Division of General Surgery, Department of Surgery, Faculty of Medicine Siriraj Hospital, Mahidol University, 12thfloor Siamindra Building, 2, Wanglang Road, Bangkok Noi, Bangkok, 10700 Thailand; 2 Department of Anatomy, Faculty of Medicine Siriraj Hospital, Mahidol University, Bangkok, Thailand

**Keywords:** COVID-19, Surgical smoke, Particle count, Contamination, Laparoscopic surgery, Smoke evacuator

## Abstract

**Background:**

The SARS-CoV2 virus has been identified in abdominal cavity of the COVID-19 patients. Therefore, the potential viral transmission from any surgical created smoke in these patients is of concern especially in laparoscopic surgery. This study aimed to compare the amount of surgical smoke and surgical field contamination between laparoscopic and open surgery in fresh cadavers.

**Methods:**

Cholecystectomy in 12 cadavers was performed and they were divided into 4 groups: laparoscopic approach with or without smoke evacuator, and open approach with or without smoke evacuator. The increased particle counts in surgical smoke of each group were analyzed. In the model of appendectomy, surgical field contamination under ultraviolet light and visual contamination scale between laparoscopic and open approach were compared.

**Results:**

Open cholecystectomy significantly produced a greater amount of overall particle sizes, particle sizes < 5 μm and particle sizes ≥ 5 μm than laparoscopic cholecystectomy (10,307 × 10^3^ vs 3738 × 10^3^, 10,226 × 10^3^ vs 3685 × 10^3^ and 81 × 10^3^ vs 53 × 10^3^ count/m^3^, respectively at p < 0.05). The use of smoke evacuator led to decrease in the amount of overall particle sizes of 58% and 32.4% in the open and laparoscopic chelecystectomy respectively. Median (interquatile range) visual contamination scale of surgical field in open appendectomy [3.50 (2.33, 4.67)] was significantly greater than laparoscopic appendectomy [1.50 (0.67, 2.33)] at p < 0.001.

**Conclusions:**

Laparoscopic cholecystectomy yielded less smoke-related particles than open cholecystectomy. The use of smoke evacuator, abeit non-significantly, reduced the particles in both open and laparoscopic cholecystectomy. Laparoscopic appendectomy had a lower degree of surgical field contamination than the open approach.

## Introduction

The Coronavirus disease 2019 (COVID-19) pandemic, caused by Severe Acute Respiratory Syndrome Coronavirus 2 (SARS-CoV-2), has been affecting our global health-care system. The SAR-CoV-2 spreads mainly through the respiratory droplets (the particles that are greater than 5 μm) produced by coughing and sneezing [1, 2]. Nevertheless, another potential mode of transmission as aerosols (those are smaller than 5 μm) could not be excluded [2, 3].

Most surgical procedures create aerosols in the form of surgical smoke through the use of various heat generating devices such as electrocautery, ultrasonic scalpels, bipolar and laser [4, 5]. Many surgical societies have recommended the use of smoke evacuator during the surgery procedure to reduce the level of surgical smoke that the surgical team is exposed to [6–9]. Surgical smoke has been established as a potential chemical hazard as it contains carcinogens as well as bacterial and viral particles [4, 10]. The SARS-CoV-2 has been identified not only in the respiratory system but also in the gastrointestinal system, blood and peritoneal fluid of the COVID19 patients [7, 11–13]. Therefore, potentially infectious surgical smoke may be produced during the abdominal operations and pose health risks to the surgical personnel [14].

In the era of COVID-19 pandemic, the best surgical approach (open versus minimally invasive approach) is still debated. This study therefore aims to compare the amount of surgical smoke and surgical field contaminations between laparotomy and laparoscopy in common surgeries such as cholecystectomy and appendectomy with or without smoke evacuator in fresh cadaveric model.

## Materials and methods

### Subjects

**Fig. 1:**
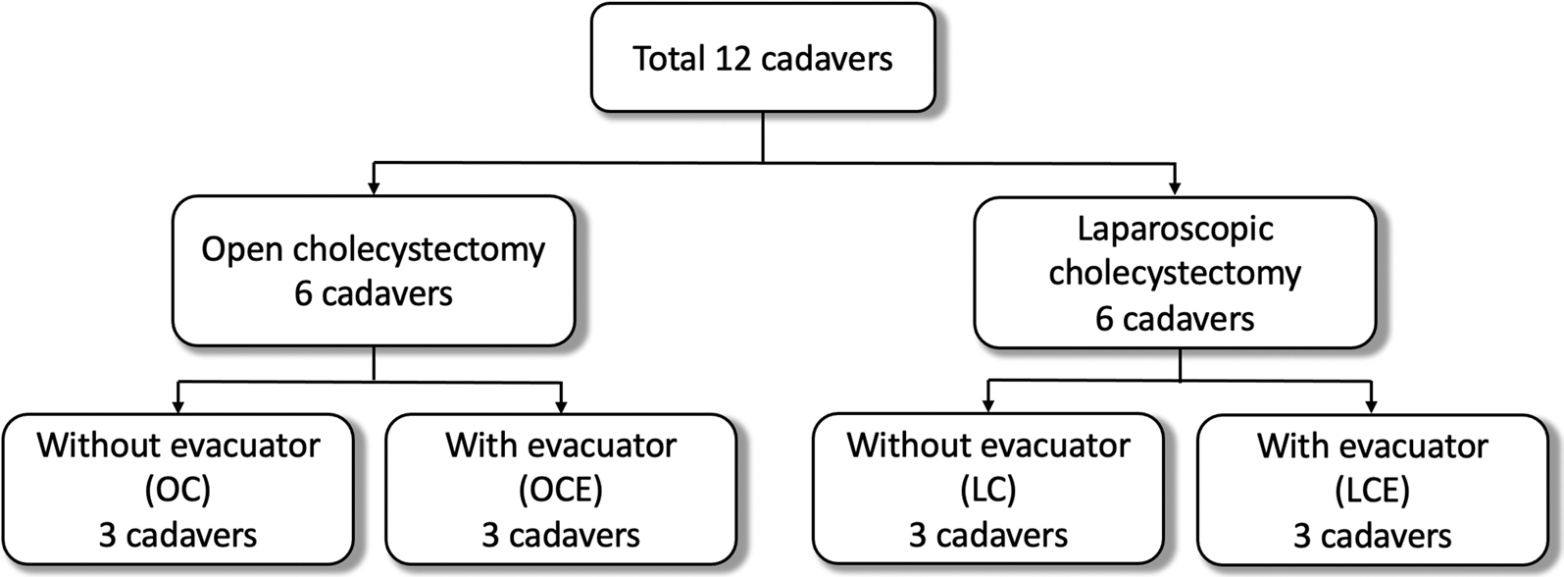
The subjects of this study (laparoscopic cholecystectomy—open cholecystectomy). *OC* open cholecystectomy without evacuator use, *OCE* open cholecystectomy with evacuator use, *LC* laparoscopic cholecystectomy without evacuator use, *LCE* laparoscopic cholecystectomy with evacuator use

This study was approved by Institutional Review Board (IRB) and was conducted at Siriraj cadaveric laboratory between August 2020 and December 2020. Total 12 fresh cadavers from the courtesy of the department of Anatomy, Faculty of Medicine Siriraj Hospital were included. All the cadavers were screened and confirmed to be safe for the study by Siriraj cadaveric protocol. Cholecystectomy was performed in the 12 subjects which were equally divided into 4 groups: laparoscopic cholecystectomy (LC), laparoscopic cholecystectomy with smoke evacuator (LCE), open cholecystectomy (OC), and open cholecystectomy with smoke evacuator (OCE) (Fig. 1). These cadaveric models were also used in the evaluation of surgical field contamination during laparoscopic and open appendectomy.

### Operative setup

**Fig. 2:**
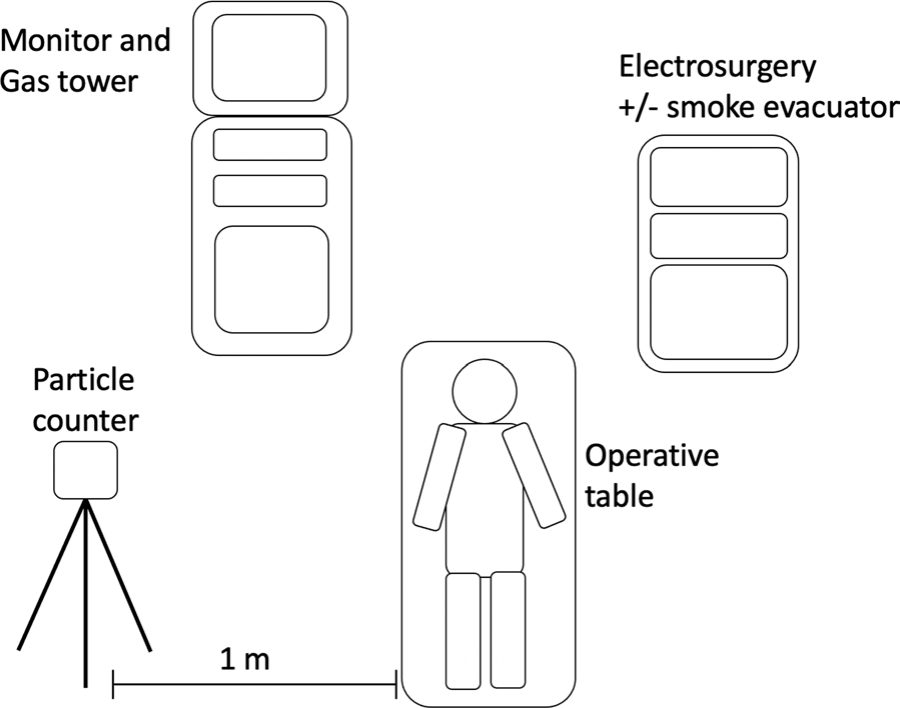
The operative setup

The operation was divided into two phases. In the first phase, cholecystectomy was performed under the four different settings (LC, LCE, OC and OCE). The number concentration of particles (particle counts, PC) in the generated surgical smoke under the four different settings were measured. In the second phase, appendectomy was performed and the degree of contaminations on the surgical field and the surgical team was detected using fluorescent-staining substance under ultraviolet light observation. All surgeries were performed in the same operative room and environment as well as by the same surgical team. The operative setup is illustrated in Fig. 2. The size of operating room was 6.0 × 7.0 × 3.2 m (width × length × height) with airflow changes of 22 times per hour.

TSI AeroTrak® (9306-04) handheld particle counter was used to measure the number concentration of particles within the surgical smoke in the diameter size range of 0.3, 0.5, 1, 3, 5 and 10 μm after adequate calibration. This standard device is mobile and normally used in monitoring of the particle counts for workers via the particle channels that provided by Division of Occupational Health. The particle counter was placed at 1 m away from an operative table on the opposite side to the primary surgeon that represented the staffs in surgical field. All particle sizes were measured and recorded every single minute during the entire operation. The PC of surgical key-steps including baseline level before commencing an operation, abdominal wall opening, gallbladder dissection, specimen retrieval, and abdominal wall closure were also noted.

Monopolar electrocautery with the same coagulation power setting at 25 Watts was used in every operation. The RapidVAC™ smoke evacuator system with Ultra-low Particulate Air (ULPA) Filter (Medtronic®) with the same suction pressure was used in OCE and LCE group. In addition to smoke evacuator, a monopolar cautery with Valleylab™ smoke evacuation pencil (Medtronic®) was used in OCE and the Valleylab™ laparoscopic smoke evacuation system (Medtronic®) was used in LCE.

Figure 2 revealed the identical experiment setup among all experiments in the cadaveric laboratory operating room. The particle counter was placed 1 m away from the operative table in the right side. In case of laparoscopic surgery, the monitor and laparoscopic system was placed additionally.

### Surgical steps of cholecystectomy

For open cholecystectomy, a 20-cm right subcostal incision was done and the abdominal wall was opened layer by layer using monopolar cautery until the abdominal cavity was reached. Surgical smoke was continuously sucked as much as possible by conventional closed suction system as our routine daily practice for open cholecystectomy. However, in OCE group, the smoke evacuation system was activated simultaneously as a monopolar cautery with smoke evacuation pencil was used. Once the gallbladder was identified, it was dissected from the gallbladder bed by Fundus-down technique and the cystic duct was clipped and cut. The specimen was then removed. As part of the preparation for the evaluation of surgical field contamination during subsequent open appendectomy, a 50 mL of fluorescent dye was sprayed at the peri-appendiceal area (appendix, right para-colic gutter and pelvic cavity). Finally, the abdominal wall was closed by 1–0 Nylon.

For laparoscopic cholecystectomy, a 12-mm infra-umbilical incision was made and a 12-mm. Covidien® optical trocar was inserted into the abdomen under direct visualization of 10-mm, 0-degree, telescope. Then, CO_2_ pneumoperitoneum was created and the intraabdominal pressure was maintained at 12 mmHg. Another three additional 5 mm. working ports were placed in the right subcostal area with a 5-mm incision (as the standard 4-ports laparoscopic cholecystectomy fashion). Once the gallbladder was found and the Calot triangle was identified, the gallbladder infundibulum was dissected using a monopolar cautery. The cystic duct was clipped and divided. Then, the gallbladder was dissected out of the gallbladder bed. Notably, in LC group, the surgical smoke occurred during the operation was intermittently sucked by conventional closed suction system. Meanwhile, in the LCE group, the surgical smoke was intermittently removed by a laparoscopic smoke evacuation system and was subsequently filtered by smoke evacuator. To prepare for the evaluation of surgical field contamination during subsequent laparoscopy appendectomy, a 50 mL of fluorescent dye was sprayed at the peri-appendiceal area as described previously. All specimens were removed through the camera port after the insufflation was discontinued and the pneumoperitoneum was completely released. Finally, all three working ports were closed by 1–0 Nylon.

### Surgical steps of appendectomy

Before performing the appendectomy, all members of surgical team wore standard full personal protective equipment (PPE) which consisted of gloves, boots, face-shield, and fully-covered waterproof suite. For open appendectomy, a 10-cm transverse skin incision was made and the abdominal wall was opened layer by layer. Once the appendix was identified, the mesoappendix was ligated by 3–0 Silk and divided until the base of appendix was reached. Then, it was ligated and divided. The appendix was removed accordingly. Finally, the abdominal wall was closed by 1–0 Nylon.

For laparoscopic appendectomy, the infraumbilical camera port from previous laparoscopic cholecystectomy was used and 12-mmHg pneumoperitoneum was maintained. Two 5-mm working ports were placed at the left lower quadrant of abdomen and suprapubic area, respectively. Once the appendix was identified, the mesoappendix was clipped and divided until the base of appendix was reached. Then, it was ligated by endo-loop and the appendix was subsequently removed and brought out via the camera port. Finally, all the incisions were closed by 1–0 Nylon.

### Objectives of the study

The primary objective was to compare the increased PC (total PC during the operation deducted by baseline PC) between laparoscopic and open cholecystectomy under the 4 subgroups (LC, LCE, OC and OCE)—using TSI AeroTrak® (9306-04) handheld particle counter. The secondary objective was to compare the surgical field contamination (the area of fluorescent dye staining under ultraviolet light) between open and laparoscopic appendectomy by using visual contamination scale (scale 0 to 10, 0 = no contamination and 10 = 100% contamination). Five key focal areas were identified for the contamination evaluation process and were as follow: skin incisions, surgical drapes, suctions, surgeon’s gloves, and face-shields. One photo of each five area was taken after completing an operation—five photos for each open appendectomy and five photos for each laparoscopic appendectomy. The degree of visual contamination was evaluated by three surgeons who were not aware of the experimental group.

### Statistical analysis

Mann–Whitney *U*-test was used to test the difference in increased PC between laparoscopic and open cholecystectomy. Kruskal–Wallis test was performed to test the difference among four subgroups without adjustment for type I error due to the exploratory analysis nature of this pilot study. The visual contamination scale was assessed by three surgeons. Agreement among three surgeons was determined by intraclass correlation coefficient (ICC: 2-way random, absolute agreement, single rating). The difference of the visual contamination scale between open and laparoscopic appendectomy was also analyzed by independent sample t-test. All statistical data analysis using SPSS software version 21.

## Results

### Demographic data

There was no statistical difference in terms of age, gender, BMI, total operative time, total cautery time and baseline total particle counts (before the operation) between open and laparoscopic approach, and among the four subgroups (Table 1).

**Table 1:** Demographic data

	Subgroup study				P-value
	OC N = 3	OCE N = 3	LC N = 3	LCE N = 3	
Age, years	61.7 (3.1)	75.3 (14.8)	78.0 (11.4)	70.3 (14.0)	0.398
Gender, (male: female)	1:2	0:3	2:1	2:1	0.363
Body mass index, (kg/m^2^)	22.7 (0.3)	21.4 (1.3)	23.8 (1.6)	23.3 (0.5)	0.278
Total operative time, minutes	25.0 (4.0)	21.7 (7.6)	33.3 (11.7)	31.00 (1.0)	0.258
Total cautery time, minutes	11.0 (2.7)	8.7 (4.0)	11.0 (1.7)	10.0 (1.7)	0.617
Baseline total particle counts (before starting an operation), × 10^3^ counts/m^3^	10,793 (2227)	13,094 (1012)	12,956 (3944)	10,561 (3088)	0.369

Data was presented as mean (standard deviation, SD) or number. *OC* open cholecystectomy without smoke evacuator use, *OCE* open cholecystectomy with smoke evacuator use, *LC* laparoscopic cholecystectomy without smoke evacuator use, *LCE* laparoscopic cholecystectomy with smoke evacuator use

### The increased particle counts between open and laparoscopic cholecystectomy

**Fig. 3:**
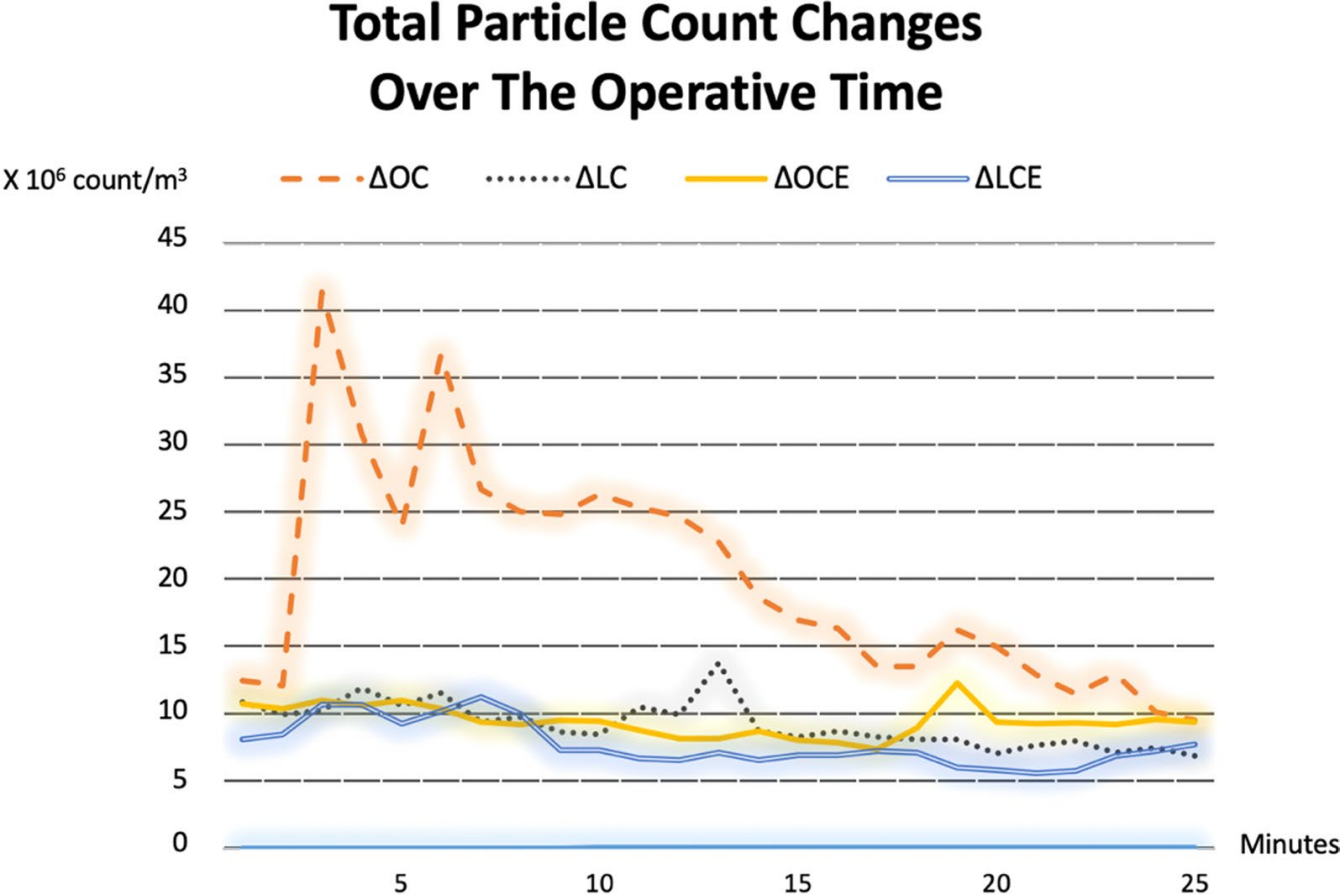
The dynamic changes in total particle counts along the operation of all subgroups. *OCE* open cholecystectomy with smoke evacuator use, *LC* laparoscopic cholecystectomy without smoke evacuator use, *LCE* laparoscopic cholecystectomy with smoke evacuator use

The increased PC of overall particle sizes, particle sizes < 5 μm and particle sizes ≥ 5 μm in open approach were significantly higher than laparoscopic approach (Table 2). In our subgroup analysis, although there was no statistical difference, the increased PC during the operation of OC and OCE was greater than LC and LCE, respectively. Moreover, smoke evacuator non-significantly reduced the increased PC during the operation in both open and laparoscopic cholecystectomy (Table 3). The dynamic changes in total PC during the operation of all subgroups are shown in Fig. 3.

**Table 2:** Comparison of the increased particle counts along the operation between open and laparoscopic cholecystectomy

	Open surgery (N = 6)	Laparoscopic surgery (N = 6)	P-value
	Increased particle counts along the operation (× 10^3^ counts/m^3^)		
Overall particle sizes	10,307 (6366)	3738 (1666)	0.009*
*Particle sizes < 5 µm*	10,226 (6358)	3685 (1670)	0.015*
0.3 µm	7243 (5912)	1996 (1156)	0.026*
0.5 µm	2060 (476)	1170 (537)	0.041*
1 µm	762 (186)	422 (110)	0.004*
3 µm	161 (33)	97 (27)	0.004*
*Particle sizes ≥ 5 µm*	81 (16)	53 (15)	0.015*
5 µm	67 (13)	42 (11)	0.002*
10 µm	14 (4)	11 (4)	0.093

Data are presented as mean (SD), *m* meter, *µm* micron

* p < 0.05

**Table 3:** Comparison of the increased particle counts along the operation among subgroups

	OC	OCE	LC	LCE	% Change between group and (p-value from pairwise comparison)			
					OC vs LC	OCE vs LCE	OC vs OCE	LC vs LCE
*Increased particle counts along the operation (× 10*^*3*^* counts/m*^*3*^*)*								
Overall particle sizes	14,516 (6774)	6099 (1512)	4461 (919)	3015 (2128)	69.3% (0.10)	50.6% (0.20)	58.0% (0.10)	32.4% (0.40)
Particles size < 5 µm	14,429 (6769)	6024 (1503)	4400 (923)	2971 (2142)	69.5% (0.10)	50.7% (0.20)	58.3% (0.10)	32.5% (0.40)
Particles size ≥ 5 µm	87 (19)	75 (14)	61 (4)	44 (18)	29.9% (0.10)	41.3% (0.20)	13.8% (0.40)	27.9% (0.70)

Data were presented as mean (SD) or percentage (p-value), *OC* open cholecystectomy without smoke evacuator use, *OCE* open cholecystectomy with smoke evacuator use, *LC* laparoscopic cholecystectomy without smoke evacuator use, *LCE* laparoscopic cholecystectomy with smoke evacuator use, *µm* micron

### Comparison of surgical field contamination between open and laparoscopic appendectomy

**Fig. 4:**
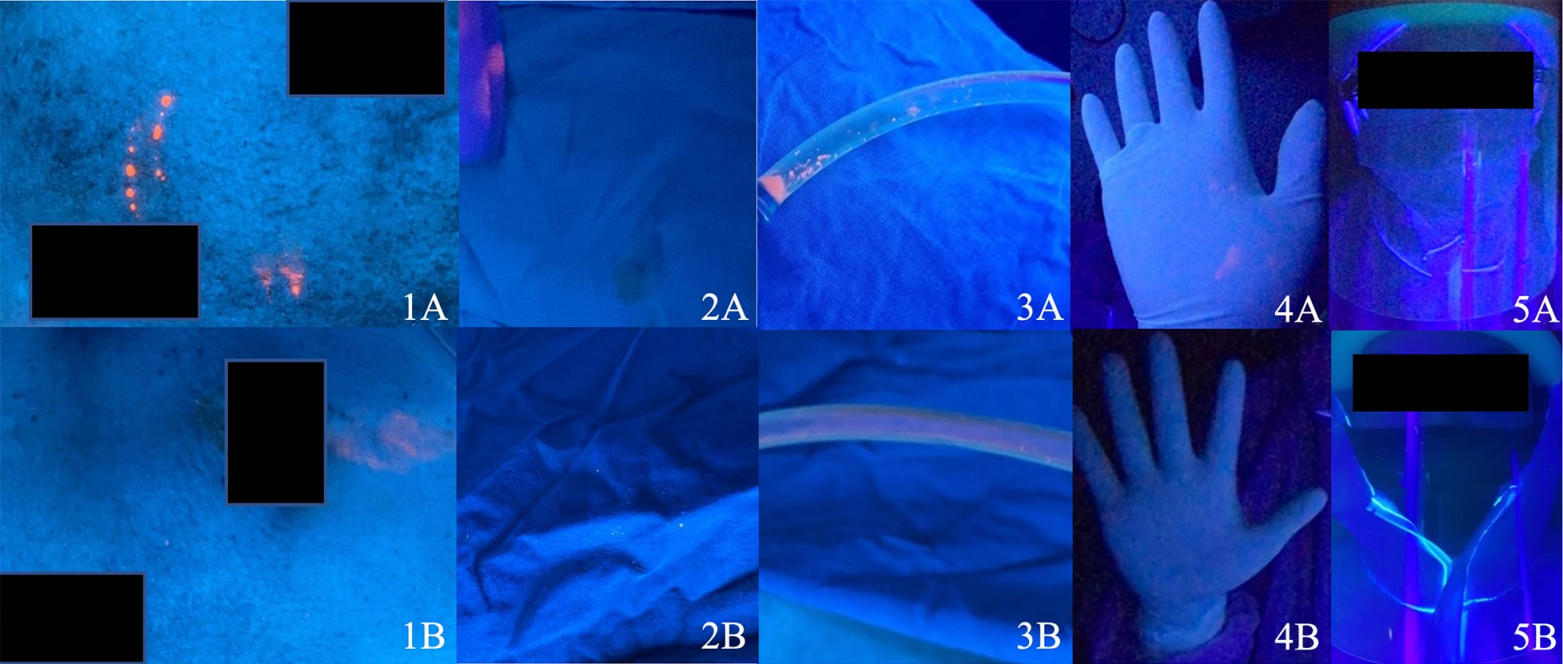
Contaminated surgical areas in open versus laparoscopic appendectomy. The examples of photographs in the blinded-questionnaires consisted of 5 areas, skin incisions (1), surgical drapes (2), suctions (3), gloves (4), and face shields (5), respectively. The upper row (**A**) was from open appendectomy and the lower row (**B**) from laparoscopic appendectomy

The degree of contamination was evaluated by three surgeons and the reliability analysis revealed intraclass correlation coefficient 0.815. The median (interquartile range) visual contamination scale in open approach [3.50 (2.33, 4.67)] was significantly greater than laparoscopic approach [1.50 (0.67, 2.33)] at p < 0.001 level. Representative photos of key areas including skin incisions, surgical drapes, suctions, surgeon’s gloves, and surgeon’s face shield between open and laparoscopic appendectomy are shown in Fig. 4.

## Discussion

This cadaveric study demonstrated that surgical smoke generated during the open cholecystectomy contained a greater number of particles (in all sizes) compared to the number of particles measured in the surgical smoke generated during the laparoscopic approach. The use of smoke evacuator non-significantly reduced the particle counts in both open and laparoscopic cholecystectomy. The degree of contamination of the surgical field and the surgical team experienced in the open appendectomy was visually higher than the contamination observed during laparoscopic appendectomy.

Some messenger ribonucleic acid (mRNA) virus such as Hepatitis B Virus (HBV), Human Immunodeficiency Virus (HIV), and Human papilloma Virus (HPV) has been detected in surgical smoke when an operation is performed in an infected individual or in a carrier [15, 16]. In addition, laryngeal papillomatosis has been found in physicians after treating HPV-related patients by laser or electrocautery suggesting that the virus can be transmitted via surgical smoke and infected closed medical personnel [17, 18]. SAR-CoV-2, another mRNA virus with a comparable size to HBV, HIV and HPV, could potentially be present in surgical smoke which contain various particle size ranging from 0.07 to 6.5 μm depending on the electrocautery device [6]. Currently, there was no direct evidence of SARS-Cov-2 virus in surgical smoke and peritoneal fluid in the study from laparoscopic appendectomy of asymptomatic COVID-19 patients by real time polymerase chain reaction (RT-PCR) [19]. In fact, the current mode of SAR-CoV-2 transmission is via droplets (particles greater than 5 µm) but another potential mode of transmission is via aerosols (particles smaller than 5 µm) [2] It is therefore highly probable that there is a risk of COVID-19 transmission to the surgical team via exposure to surgical smoke generated during surgery on infected individuals. The minimization of surgical smoke is critical to lower the risk of COVID-19 transmission to the surgical team.

Both cholecystectomy and appendectomy were chosen for this study because they are simple and common abdominal surgical procedures in the world. Generally, the laparoscopic approach yields more benefits such as less postoperative pain, less surgical scar, better recovery period, shorter hospital stays and less wound-related complications compared to open approach. However, the best surgical approach during the COVID-19 pandemic is still being debated. The non-operative management should be considered first, particularly infected patients. Preoperative risk assessment and nasopharyngeal swab by RT-PCR for SARS-Cov-2 virus should be performed in all operative cases if possible. If the emergency operation is needed, all protective maneuvers must be applied to all operative staffs [6, 7]. Those who support the open approach suggest that the surgical smoke and contamination created during surgery is more controllable compared to the laparoscopic approach because there is no concern about gas leak from pneumoperitoneum creation. On the contrary, those who support laparoscopic approach advise that the generated surgical smoke should be easier to control because the operation was performed in the abdominal cavity as a closed space and it could be simply evacuated out of the abdomen by conventional closed suction [6, 9]. Until now, there were very few studies which compared the amount of surgical smoke generated between laparoscopic and the open approach. The results from these studies were heterogeneous. Li et al. revealed that the particle concentration reached maximally at 10 min after electrosurgery and the particle counts of 0.3- and 0.5-micron-particle were increased in laparoscopic surgery than in open surgery [5]. In contrast, Mintz et al. reviewed narratively and suggested laparoscopy over laparotomy in reduction of surgical smoke due to close space that the adequate gas control by a number of maneuvers can be applied [20]. The possible reason for the inconclusive results is the difference of operative set up and environment amongst the studies. Therefore, in this study, every operation was performed under the same settings to reduce the factors that might interfere the results.

In this study, the open approach generated more surgical smoke. This finding is in agreement with a recent study conducted by Kameyama et al. They concluded that open approach produced more surgical smoke than laparoscopic approach in colorectal surgery [21]. However, to perform laparoscopic surgery during this era, the surgical teams must follow the recommendations strictly, included smallest incision to prevent gas leak, avoidance of hand-assisted surgery, low flow and pressure of pneumoperitoneum, use of closed-suction system, preferred sharp dissection, total removal of gas closely, etc. [6, 7]. Another interesting finding of this study is that applying the smoke evacuator decreased the surgical smoke non-significantly in both open and laparoscopic cholecystectomy. By using this device, the increased PC of overall particle sizes of open and laparoscopic cholecystectomy were reduced (about 58% and 32% reduction, respectively at p > 0.05). Of the four groups, the average increased PC during the operation of OC was the highest. The maximum level of measured PC during OC corresponds to abdominal opening (first 5 min) when the energy device was used the most (Fig. 3). The use of the smoke evacuator, to reduce the surgical smoke may therefore be more relevant to open cholecystectomy.

In the surgical field contamination evaluation, fluorescent dye under ultraviolet light was used because the technique is uncomplicated, and it could simply demonstrate both visible and invisible contaminated area by illuminating under ultraviolet light. In addition, five key areas were selected because they were commonly contaminated disposable areas which the surgical personnel must be aware while discarding them. Interestingly, the degree of visual contamination of the surgical field and the surgical team in the open approach was greater than the laparoscopic approach. The subjective evaluation of the surgical field contamination was undertaken by an independent panel of three surgeons who were not aware of the experimental group to minimize any individual biasness. As a result, it could be assumed that laparoscopic surgery under standard protection may be done safely without increasing the degree of contamination and it should be considered during this pandemic.

To the best of our knowledge, this is the first study to be conducted which compares the generated surgical smoke between open and laparoscopic surgery with or without smoke evacuator use in the fresh cadaveric model. This model was perceived to be safer to study than live human particularly in this pandemic and the findings are reliable because all attempts have been made to eliminate all the potential factors that may interfere with the results. However, it is acknowledged that there are some limitations to this pilot exploratory study such as the sample size i.e., number of operations, the potential differences in the tissue characteristic between the fresh cadaver and the live human and the viral components within the surgical smoke. From our observation, gross tissue characteristics of fresh cadavers were closed to the human, however, there was no bleeding from procedures. There are current plans to undertake further studies to better elucidate the findings of this pilot study by using a larger randomized clinical trial and to compare the true viral components in surgical smoke between open and laparoscopic approach of more complex surgeries and the benefit of smoke evacuator.

## Conclusions

Laparoscopic cholecystectomy generated less surgical smoke than open cholecystectomy in this study. Using smoke evacuator allowed non-significant reduction of the smoke particles in both open and laparoscopic cholecystectomy and could be considered in any surgery. Furthermore, laparoscopic appendectomy carried a lower degree of surgical field contamination than the open approach. Nevertheless, randomized clinical trials are needed to prove the true advantages of laparoscopic over open surgery as well as surgical smoke evacuator system.

## Data Availability

The datasets used and/or analysed during the current study are available from the corresponding author on reasonable request.

## Abbreviations

cm: Centimeter
COVID-19: Coronavirus disease 2019
Et al.: Et alibi, and elsewhere
HBV: Hepatitis B virus
HIV: Human immunodeficiency virus
HPV: Human papilloma virus
ICC: Intraclass correlation coefficient
IQR: Interquatile range
IRB: Institutional Review Board
i.e.: Id est, in other words
LC: Laparoscopic cholecystectomy without evacuator use
LCE: Laparoscopic cholecystectomy with evacuator use
m: Meter
mL: Milliliter
mm: Millimeter
mRNA: Messenger ribonucleic acid
OC: Open cholecystectomy without evacuator use
OCE: Open cholecystectomy with evacuator use
PC: Particle count
PPE: Personal protective equipment
SARS-CoV-2: Severe Acute Respiratory Syndrome Coronavirus 2
SD: Standard deviation
ULPA: Ultra-low particulate air
UV: Ultraviolet
Vs: Versus
µm: Micron
